# Lessons from *Toxoplasma*: Host responses that mediate parasite control and the microbial effectors that subvert them

**DOI:** 10.1084/jem.20201314

**Published:** 2021-10-20

**Authors:** Eva-Maria Frickel, Christopher A. Hunter

**Affiliations:** 1 Institute of Microbiology and Infection, School of Biosciences, University of Birmingham, Edgbaston, UK; 2 Department of Pathobiology, School of Veterinary Medicine, University of Pennsylvania, Philadelphia, PA

## Abstract

The intracellular parasite *Toxoplasma gondii* has long provided a tractable experimental system to investigate how the immune system deals with intracellular infections. This review highlights the advances in defining how this organism was first detected and the studies with *T. gondii* that contribute to our understanding of how the cytokine IFN-γ promotes control of vacuolar pathogens. In addition, the genetic tractability of this eukaryote organism has provided the foundation for studies into the diverse strategies that pathogens use to evade antimicrobial responses and now provides the opportunity to study the basis for latency. Thus, *T. gondii* remains a clinically relevant organism whose evolving interactions with the host immune system continue to teach lessons broadly relevant to host–pathogen interactions.

## Teaser

*Toxoplasma gondii* is a clinically relevant organism that teaches lessons broadly relevant to host–pathogen interactions. This review highlights how this organism is detected and how the cytokine IFN-γ promotes control of vacuolar pathogens. This article also evaluates the diverse strategies used by *T. gondii* to evade antimicrobial responses and develop latency.

## *Toxoplasma gondii*: A pathogen and model organism

The lifecycle of the intracellular protozoan *T. gondii* is complex, but infection of any warm-blooded animal is characterized by an acute phase during which the replication of the dominant tachyzoite stage leads to parasite spread. As the immune response develops, this developmental stage is controlled, but the parasite converts to the slow-growing bradyzoite present in long-lived tissue cysts in the brain and muscle. In an immunocompetent host, this infection is typically regarded as persistent but asymptomatic, but *T. gondii* is a life-threatening opportunist in hosts with defects in cell-mediated immunity. However, even in immunocompetent individuals, the ability of the parasite to infect and lyse diverse cell types contributes to its ability to cause disease. Because *T. gondii* infects mice, this natural host–pathogen combination provides a model to understand (1) how the host can sense infection; (2) the events that lead to the production of cytokines (IL-12 and IL-1 family members) that promote the production of IFN-γ; and (3) the IFN-γ–mediated pathways that allow infected cells to limit parasite replication. In particular, the ability of IFN-γ to activate immune and nonimmune cells to restrict *T. gondii* growth has provided a tractable system to identify the host effector pathways that are important to cope with vacuolar pathogens. Nevertheless, despite an array of host mechanisms that limit the replication of *T. gondii*, multiple parasite-derived factors that subvert host cell activities allow this organism to survive in disparate cell types and hosts. A key element for this success is that, as an apicomplexan, *T. gondii* has specialized secretory organelles, the rhoptries (ROPs) and dense granules (GRAs), that are used to secrete effector molecules into the host cell. This review focuses on recent advances in our understanding of the interactions between parasite-derived effectors, the host cell, and the immune system that determine the outcome of infection.

## Innate detection of *T. gondii*

A key principle that underlies resistance to infection is the ability of pattern recognition receptors to sense microorganisms and direct the development of protective immunity. The ability of *T. gondii* to invade and replicate in a host cell involves injection of ROP effector proteins, the creation of a parasitophorous vacuole (PV), and the secretion of GRA proteins into the host cytosol and parasite growth. These processes result in exposure of parasite material to the host and cause many changes in host cells associated with immune and cellular stress pathways (Rastogi et al., 2020), but their impact on innate recognition of *T. gondii* is not well understood. In murine models, the ability of *T. gondii* to induce dendritic cell (DC) and macrophage production of IL-12 suggested a mechanism to directly recognize parasite-derived molecules. Indeed, *T. gondii* strains differ in their ability to induce IL-12, a property linked to the parasite GRA proteins, GRA15 (Rosowski et al., 2011) and GRA24 (Braun et al., 2013; Mercer et al., 2020). The interpretation of these types of observations is a challenge: It is unclear whether a host sensor that detects these GRAs induces IL-12 or whether GRA-mediated alterations of the host cell trigger cytokine production. The latter possibility relates to the broad concept that infection-induced perturbations of host cell functions trigger host responses that are agnostic to the specific pathogen (Lopes Fischer et al., 2020).

### A role for TLRs

**Figure 1.**
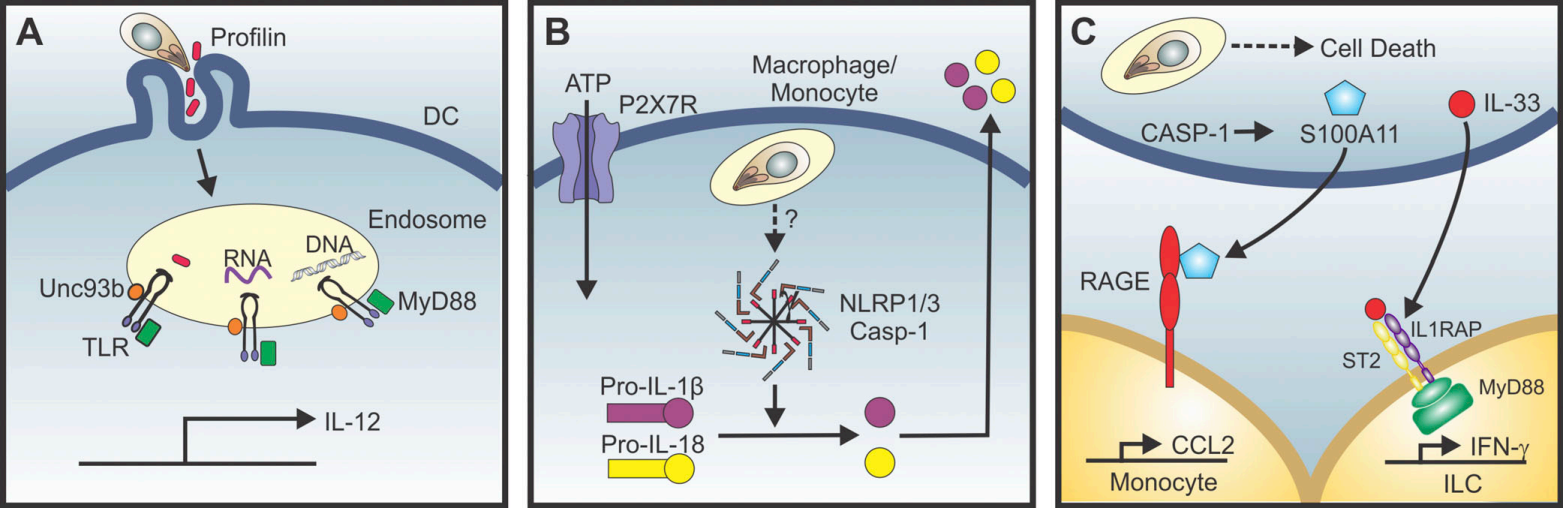
**Pathways of *T. gondii* sensing.** **(A)** TLR-dependent sensing of *T. gondii* products in murine dendritic cells leads to the production of IL-12. **(B)** P2X7R is associated with inflammasome-driven (NRLP1/3) processing of IL-1β and IL-18 and is dependent on TLR4 stimulation in murine bone marrow–derived macrophages but constitutively active in human monocytes. **(C)** The death of *T. gondii*–infected cells results in the release of S100A11, which induces monocyte CCL2 production, while the release of IL-33 promotes ILC production of IFN-γ. RAGE, receptor for advanced glycation end products.

TLRs are germ line–encoded pattern recognition receptors that recognize pathogen-associated molecular patterns (PAMPs) and are critical for resistance to many infections. These sensors recognize a range of PAMPs and use the signaling adaptors MyD88 and TRAF6 to activate NF-κB and MAPK signaling, which promote chemokine and cytokine production. In mice, MyD88 and UNC93B (an ER-resident protein associated with trafficking of endosomal TLR3, 7, and 9) contribute to IL-12 production and resistance to *T. gondii* (Melo et al., 2010; Pifer et al., 2011). The identification of the parasite molecule profilin as a PAMP recognized by murine TLR11/12 connected MyD88 dependence and TLR-mediated recognition of *T. gondii* (Andrade et al., 2013; Koblansky et al., 2013; Yarovinsky et al., 2005; Fig. 1 A). However, TLR11 and 12 are not functional in humans (Yarovinsky, 2014), and in mice, the description of MyD88-independent pathways to control *T. gondii* indicates the relevance of additional mechanisms involved in recognition of *T. gondii* (López-Yglesias et al., 2019; Mercer et al., 2020; Mukhopadhyay et al., 2020; Sukhumavasi et al., 2008).

### *T. gondii*–mediated activation of the inflammasome

The Nod-like receptor (NLR) family members are cytosolic molecules that can bind PAMPs and nucleate the assembly of the inflammasome complex, which in turn leads to activation of proteases that amplify inflammatory signals. Relevant to toxoplasmosis, this process engages the adapter protein apoptosis-associated speck-like protein containing a CARD (ASC), which results in caspase-1–mediated processing of IL-1α, IL-1β, and IL-18 to their bioactive forms, but can also lead to pyroptosis, a form of inflammatory cell death (Snyder and Oberst, 2021). Genome-wide association studies linked single nucleotide polymorphisms in the N-terminus of NLRP1 with susceptibility to congenital toxoplasmosis (Witola et al., 2011) and the NLRP3 inflammasome activator P2X7R with clinical toxoplasmosis in immunocompetent patients (Lees et al., 2010). In contrast, polymorphisms in the NLRP1 locus of Lewis rats are associated with enhanced death of cells infected in vitro and increased resistance to *T. gondii* in vivo (Cavaillès et al., 2006; Cirelli et al., 2014). The eight amino acid sequence in the N-terminus of Lewis rat NLRP1 responsible for conferring *T. gondii* resistance is not cleavable by anthrax lethal toxin, which is a known activator of NLRP1. The parasite GRA proteins GRA35, GRA42, and GRA43 have recently been shown to contribute to the activation of the Lewis rat NLRP1 and pyroptosis, but as no direct interaction between these GRA proteins and NLRP1 could be detected, the mechanismn of activation is elusive (Wang et al., 2019b). Human NLRP1 in its N-terminus also does not contain a lethal toxin cleavage motif but does have a pyrin domain to mediate ASC association. Knockdown of NLRP1 in human macrophages protected against long-term cell death in infected cultures, but not over the short time span of hours associated with pyroptotic cell death (Witola et al., 2011). Hence, the precise role of NLRP1 action in human macrophages remains to be defined.

In murine macrophages, NLRP1 and NLRP3 are implicated in the detection of *T. gondii* and subsequent production of IL-1β and IL-18 (Ewald et al., 2014; Gorfu et al., 2014; Fig. 1 B). Likewise, infection of human monocytes with *T. gondii* activates NLRP3, which results in release of IL-1β, but without pyroptosis (Gov et al., 2017; Pandori et al., 2019). Although endogenous IL-1β and IL-18 appear to have a limited role in resistance to *T. gondii* (Park and Hunter, 2020), in the brain, the ability of microglia to release IL-1α contributes to parasite control (Batista et al., 2020). While the majority of studies on the inflammasome have revolved around the interactions of *T. gondii* with macrophages, other studies highlight the ability of *T. gondii* to interfere with the inflammasome pathway and block the activation of proapoptotic caspases to extend the lifespan of infected human neutrophils (Lima et al., 2018; Lima et al., 2021). The inflammasome and other caspase-containing complexes that are activated during *T. gondii* infection have additional functions beyond the processing of IL-1 family members. For example, the ability of IFN-γ–primed human macrophages to kill *T. gondii* leads to release of parasite DNA into the cytosol, where it can be sensed by AIM2 to drive host cell apoptosis without IL-1β production in a caspase-8–dependent fashion (Fisch et al., 2019a; Fisch et al., 2020). The enzymatic activity of caspase-8 is also required for activation of the NF-κB member c-Rel for IL-12 production and resistance to *T. gondii* (DeLaney et al., 2019).

Despite reports that infection with *T. gondii* leads to the activation of NLRs, there is currently no evidence that infected cells directly detect a parasite product via NLRs. This raises the question, how does cell-intrinsic innate sensing of *T. gondii* occur? Intriguingly, uninfected bone marrow–derived macrophages undergo spontaneous NLRP1 activity when treated with inhibitors of the host serine protease DPP8/9. This observation suggests that DPP8/9 limits the accumulation of a “self” signal detected by NLRP1 and any process that interferes with levels or activity of this protease would provide a mechanism to detect cellular perturbations. The sensitivity of various rat strain macrophages to undergo pyroptosis in response to the DPP8/9 inhibitor phenocopies *T. gondii*–induced pyroptosis in these strains. This has led to the proposal that host cells infected with *T. gondii* may use a similar pathway to activate NLRP1 (Gai et al., 2019), an idea that has yet to be formally tested.

### A role for alarmins

Many of the datasets described above are viewed in the context of models in which cells infected with *T. gondii* would be directly exposed to parasite-derived material that culminates in the production of cytokines that promote cell-mediated immunity. This model is largely inconsistent with in vivo data that infected cells are not major sources of IL-12 (Christian et al., 2014). Similarly, human monocytes infected in vitro do not produce IL-12, although those that phagocytose live *T. gondii* do (Tosh et al., 2016). The ability of *T. gondii* to interfere with innate recognition in infected cells (discussed below) highlights a common theme for many pathogens that there is a need for uninfected cells to be able to respond to the presence of infection. One mechanism that addresses this problem is that inflammatory host cell death (pyroptosis, necroptosis, or mechanical) of infected cells results in the release of an array of structurally unrelated molecules associated with cellular damage that provide “danger” signals. Indeed, the replication of *T. gondii* is associated with lysis of host cells and the release of the alarmins ATP, S100A1, ISG15, and IL-33 that activate immune populations (Fig. 1, B and C). Thus, human monocytes infected with *T. gondii* activate caspase-1, which results in the release of the calcium binding protein S100A11 that activates the receptor for advanced glycation end products on bystander cells and induces CCL2 production (Safronova et al., 2019). Other examples revolve around the IL-1 family, as exemplified by P2X7R, the purinergic receptor for ATP that mediates cellular depolarization and inflammasome activation associated with cell death, IL-1 processing, and ROS production. P2X7R has been linked to intracellular killing of *T. gondii* (Lees et al., 2010); to the ability of *T. gondii* to promote epithelial cell production of CCL5, TNF, and IL-6 (Huang et al., 2017); and to NLRP3 inflammasome activation in human epithelial cells and mouse macrophages (Moreira-Souza et al., 2017; Quan et al., 2018). Likewise, ISG15 is an unconventional secreted alarmin (Perng and Lenschow, 2018), and its release at the site of infection contributes to the recruitment of DCs that produce IL-1β and enhance local production of IFN-γ (Napolitano et al., 2018). IL-33 is an IL-1 family member that does not require proteolytic processing, but because it exists preformed in the nucleus of stromal cells, its release is a consequence of cellular damage. In mice infected with *T. gondii*, parasite replication results in increased levels of IL-33, which promotes innate lymphoid cell (ILC) production of IFN-γ (Clark et al., 2021), whereas during toxoplasmic encephalitis, IL-33 acts on astrocytes (Still et al., 2020). IL-33 uses the MyD88 adapter to signal and thus contributes to the MyD88-dependent activation of ILCs required for resistance to *T. gondii* (Ge et al., 2014). The list of alarmins continues to grow, and to date, there is a select list of those that have been implicated in the response to *T. gondii*. Because this parasite can infect all nucleated cells, it seems likely that cell- and tissue-specific signals provided by alarmins will tune the magnitude of the local inflammatory response. A related question revolves around whether the ability of *T. gondii* to modulate different forms of cell death affects the release of alarmins and represents a parasite strategy to evade recognition.

## IFN-γ–mediated antimicrobial effector mechanisms

It has long been appreciated that IFN-γ can promote the respiratory burst in macrophages to limit the growth of *T. gondii* and, in nonhematopoietic cell types, can induce expression of indolamine dioxygenase (IDO), which depletes intracellular tryptophan required for parasite growth (Nathan et al., 1983; Pfefferkorn, 1984). IFN-γ is also critical for resistance to *T. gondii* in vivo (Suzuki et al., 1988), and this is a function of the ubiquitous expression of the IFN-γR and its ability to activate hematopoietic and nonhematopoietic cells to limit parasite replication in vivo (Yap and Sher, 1999). IFN-γ signaling is mediated by the transcription factor signal transducer and activator of transcription 1 (STAT1), which promotes the expression of a large number of genes collectively called IFN-stimulated genes (ISGs). Not surprisingly, the loss of STAT1 in mice mirrors the loss of IFN-γ, and these mice are highly susceptible to toxoplasmosis (Gavrilescu et al., 2004; Lieberman et al., 2004). Moreover, the lineage-specific deletion of STAT1 in macrophages or astrocytes in vivo results in decreased ISG expression and increased parasite replication (Hidano et al., 2016; Wang et al., 2019a).

**Figure 2.**
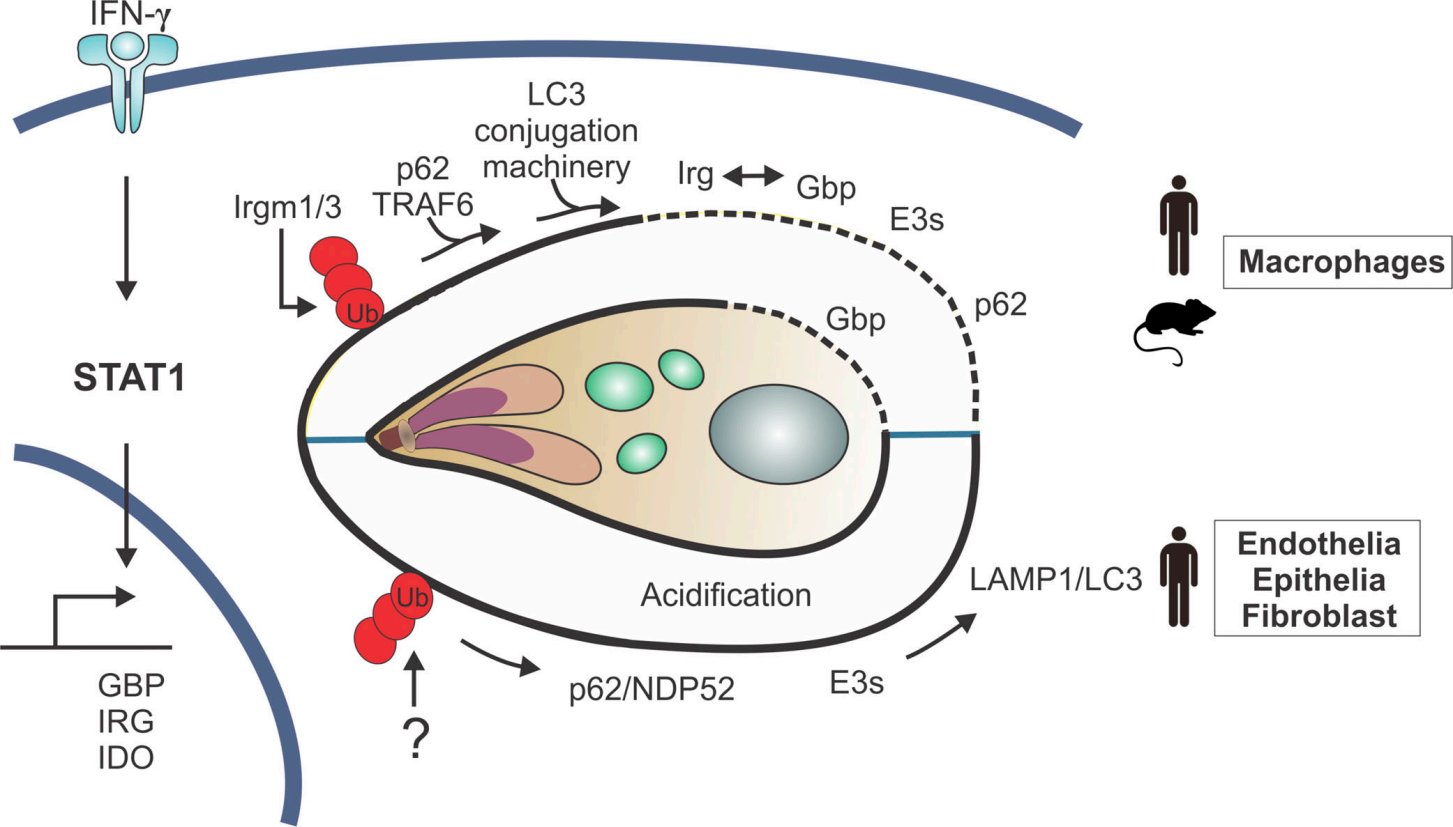
**Pathways of IFN-γ–dependent *T. gondii* elimination.** IFN-γ induces a multitude of host defense molecules via STAT1. Top: In murine cells, the *T. gondii* PVM is seeded with host defense molecules ubiquitin (Ub), Gbps, and Irgs controlled by murine Irgm1/3, the host autophagy LC3 conjugation machinery, and Traf6 and p62, which engage in a feedback mechanism. Further E3s and p62 localize to the PV, and murine Irgs and Gbps drive the disruption of the PVM and the parasite plasma membrane (a mechanism also observed in human macrophages). Bottom: Human cells control *T. gondii* by ubiquitin deposition controlled by an unknown factor and binding of p62 and NDP52. E3s participate (Traf 2 and Traf6), leading to vacuolar acidification or growth stunting of the parasite.

It is now recognized that IFN-γ induces a cascade of events that involve recognition, tagging, and disruption of the PV to expose the parasite surface to host effectors. While core processes involved in restriction of *T. gondii* are conserved between cell types and species, there are important differences. For example, in hematopoietic and nonhematopoietic murine cells and human macrophages, control of the parasite is achieved through PV breakage (Fig. 2). For human nonhematopoietic cells, numerous pathways are implicated in the events that lead to parasite control that vary with cell type and include nonacidifying autophagy, non–PV targeting GBP1-mediated control, and PV acidification (Clough et al., 2016; Johnston et al., 2016; Mukhopadhyay et al., 2020; Selleck et al., 2015). One of the major advances in this area is the recognition that the autophagy and the ubiquitin–proteasome system involved in cellular housekeeping function to tag proteins for degradation and deal with damaged organelles is also critical for IFN-γ–mediated clearance of pathogens. Conventional autophagic processes do not appear to be involved in control of *T. gondii* (Besteiro, 2019), but the ability of this machinery to recognize foreign or damaged membranes intersects with the IFN-γ–inducible large GTPases, the immunity-related GTPases (IRGs) and the guanylate-binding proteins (GBPs) to mediate parasite control. There is abundant evidence that interfering with these pathways in vivo results in increased susceptibility to *T. gondii* (Collazo et al., 2001; Degrandi et al., 2013; Foltz et al., 2017; Liesenfeld et al., 2011; Ling et al., 2006; MacDuff et al., 2015; Steffens et al., 2020; Taylor et al., 2000; Taylor et al., 2007; Yamamoto et al., 2012; Zhao et al., 2008) and the molecular pathways involved are discussed in more detail below.

### Role of autophagic processes in IFN-γ–mediated control of *T. gondii*

How IFN-γ promotes the initial recognition of the *T. gondii* PV is unclear, but it relies on ubiquitin-targeting of the PV, and the host E3 ubiquitin ligases (Trim21 and Hoil-1) have a role in resistance to *T. gondii* in vivo (Foltz et al., 2017; MacDuff et al., 2015). IFN-γ–mediated growth restriction depends on core members of the ATG (autophagy-related) proteins and the regulator of autophagosome formation termed microtubule-associated protein 1A/1B light chain 3 (LC3). The current model of selective autophagy emphasizes the importance of cargo receptors, which, by binding eat-me signals such as phosphatidylserine residues and LC3/GABARAP (γ-aminobutyric acid [GABA] receptor–associated protein) family members, achieve selectivity through juxtaposing cargo and phagophores. In human nonhematopoietic cells, the cargo receptors and autophagy adaptors, NDP52 and p62, are required for IFN-γ to control *T. gondii* (Clough et al., 2016; Selleck et al., 2015), and p62 is required for the recruitment of LC3 and GABARAPL2 to the PV (Zhang et al., 2020). The entire LC3 conjugation system has been implicated in recruitment of IRGs and GBPs to the parasitophorous vacuolar membrane (PVM) in murine cells: Atg5 (Khaminets et al., 2010), Atg3 (Choi et al., 2014; Haldar et al., 2014), Atg7, and Atg16L1 (Ohshima et al., 2014), as well as all LC3 (Atg8) homologues (Park et al., 2016). Phosphorylated products of phosphatidylinositol on the PVM may bring the Atg12-Atg5-Atg16L1 complex to the membrane via effector proteins that link phosphoinositides to the Atg complex (Park et al., 2016). Another IFN-γ–induced protein, ISG15 (described earlier as an alarmin), is analogous to ubiquitin in that its attachment to other proteins can alter localization and function, and loss of ISG15 results in impaired recruitment of p62, NDP52, and LC3 to the PV and reduced ability to control parasite growth (Bhushan et al., 2020).

### IFN-γ–induced IRGs target *T. gondii*

Another group of ISG are the murine IRGs, which are members of the dynamin superfamily, and these GTPases use the energy of GTP hydrolysis to remodel cellular membranes. Of the 23 mouse IRG proteins, studies with *T. gondii* heralded the role of Irgm1 (LRG-47), Irgm2 (GTPI), Irgm3 (IGTP), Irgd (IRG-47), Irga6 (IIGP1), Irgb6 (TGTP), and Irgb10 in resistance to intracellular infections (Collazo et al., 2001; Liesenfeld et al., 2011; Taylor et al., 2000). Irgm proteins have a GTPase motif that contains the amino acids GMS, while the other IRGs have a GKS amino acid motif. Mechanistically, the GKS IRGs bind to GTP and target the *T. gondii* PV (for in-depth discussion, see Pilla-Moffett et al. [2016]), and the ability of the GKS Irgb6 to bind specific phospholipids is involved in the recognition of the PV (Lee et al., 2020). In mice, the GMS IRGs Irgm1 and 3 render GKS IRGs inactive and are bound to endomembranes preventing GKS IRG and murine GBP mistargeting of these “self organelles,” and in one model the absence of Irgm1 from the PV allows this compartment to be targeted by effector IRGs (Haldar et al., 2013; Maric-Biresev et al., 2016). However, Irgm1 can target the phagosome (a cellular compartment distinct from the PV) of *Mycobacterium tuberculosis* (MacMicking et al., 2003; Singh et al., 2006; Tiwari et al., 2009), and Irgm2 and 3 can be recruited to the PV (Al-Zeer et al., 2009; Hunn et al., 2008; Khaminets et al., 2010; Ling et al., 2006; Martens et al., 2005; Melzer et al., 2008). Irgm2 has several activities that are involved in these processes, including the recruitment of Gbp1 and Irgb6 to the PV without itself being recruited (Pradipta et al., 2021). Thus, while Irgm1/3 have emerged as regulators of ubiquitin, IRG and GBP, and p62 targeting of the PV (Haldar et al., 2015; Fig. 2), there are open questions about this complex cascade. Nevertheless, the absence of Irgm1 promotes susceptibility to a number of intracellular infections in vivo*,* but infection of Irgm1^−/−^ mice results in a general lymphomyeloid collapse (Feng et al., 2004; Feng et al., 2008). This phenotype highlights the role of IRGs in the cellular housekeeping associated with an immune response, and the lymphomyeloid collapse is rescued by double deletion of Irgm1 and 3 (Henry et al., 2009). The finding that Irgm1/3^−/−^ animals remain susceptible to *T. gondii* while exhibiting resistance to *Salmonella* infection (Henry et al., 2009) is an example of the need to distinguish the effects of Irgms in the context of cell-autonomous pathogen control from their impact on the immune response. The observation that the repertoire of IRG genes in humans is restricted compared with rodents, and that IRGs are not regulated by IFN-γ, indicates the presence of alternative pathways to regulate ubiquitin-centric control of *T. gondii*. One consequence of the absence of functional human Irgs is that PV destruction is limited to human macrophages (see below) but happens in all murine cell types. This exposure of parasitic PAMPs in all cell types could lead to more cell death and release of alarmins, providing a mechanism to amplify the inflammatory response in the murine system.

### IFN-γ–induced GBPs as anti–*T. gondii* effectors

GBPs are IFN-γ up-regulated GTPases of the dynamin superfamily involved in the regulation of membrane, cytoskeleton, and cell cycle progression dynamics and have been linked to control of a number of intracellular bacteria and parasites (Tretina et al., 2019). Their genes are arranged in clusters on chromosomes 3 and 5 and in one cluster of seven GBPs in the human genome (Degrandi et al., 2007; Kim et al., 2011; Kresse et al., 2008; Olszewski et al., 2006). Early studies showing that IFN-γ induced GBP expression also established that murine Gbp1, 2, 3, 6, 7, and 9 were recruited to the PV of cells infected with *T. gondii* and that virulent strains interfered with recruitment (Degrandi et al., 2007). These findings foreshadowed their critical role in control of intracellular pathogens. We now know that recruitment to the PV is dependent on their GTPase activity (Virreira Winter et al., 2011), and that the ability of a multitude of murine Gbps to target the PV is subject to a recruitment hierarchy, with Gbp2 preceding Gbp7 (Kravets et al., 2016; Steffens et al., 2020). GBP targeting of the *T. gondii* PV in murine cells and human macrophages leads to vacuole breakage, the release of parasite DNA, and AIM-2–mediated activation of the inflammasome (Fisch et al., 2020; Kravets et al., 2016; Selleck et al., 2013; Yamamoto et al., 2012). Deletion of Gbps (either the cluster present on chromosome 3 or Gbp1) results in reduced GKS IRG recruitment (Selleck et al., 2013; Yamamoto et al., 2012). In human macrophages, stromal cells, and haploid HAP1 cells, GBP1 is recruited to the PV (Fisch et al., 2019a; Fisch et al., 2020; Ohshima et al., 2014; Qin et al., 2017), while in epithelial cells, GBP1 promotes parasite control but is not recruited to the PV (Johnston et al., 2016). It is thus unclear how GBPs control *T. gondii* growth from a parasite distal location. It also remains undefined what entities GBPs recognize on the PV or parasite surface, but the ability of human GBP1 to bind to damaged endomembranes, bacterial surfaces, and lipopolysaccharide structures requires the C-terminal farnesylation motif, GTPase activity, and a triple arginine stretch in GBP1 (Kutsch and Coers, 2020). These features suggest that GBP1 binds to the glycans on the luminal side of bacterial or cellular vacuole compartments (Feeley et al., 2017; Piro et al., 2017). Because human GBP1 localizes to lipid bilayers but does not break their integrity (Sistemich et al., 2020), it is likely that additional host effectors ultimately lyse the PV and the parasite plasma membrane. The contribution of individual Gbps in these processes, and their broader impact on chromosome 5 (Gbp4, 6, 8, 9, 10, and 11) and human GBPs 2–7 in the control of *T. gondii*, remain to be defined.

### IFN-γ–independent activation of parasite killing

Although IFN-γ has such a dominant role in resistance to *T. gondii* in vivo, the type I IFNs have also been implicated in parasite control (Han et al., 2014; Matta et al., 2019). There are also T cell–dependent, IFN-γ–independent pathways that contribute to parasite control, including the ability of recently activated T cells to express the surface molecule CD40 ligand. The molecule CD40 can be expressed on immune and nonimmune cells, and signals through CD40 are sufficient to stimulate accumulation of the autophagy molecule LC3 around the parasite, vacuole-lysosomal fusion, and death of *T. gondii* (Ogolla et al., 2013; Subauste, 2019). CD40 ligand is a member of the TNF superfamily of immunomodulators, and while the ability of TNF to potentiate the effects of IFN-γ has long been recognized (Sibley et al., 1991), whether other family members contribute to IFN-γ–independent activities and resistance to *T. gondii* is unclear.

## *T. gondii* effectors that subvert host responses

**Figure 3.**
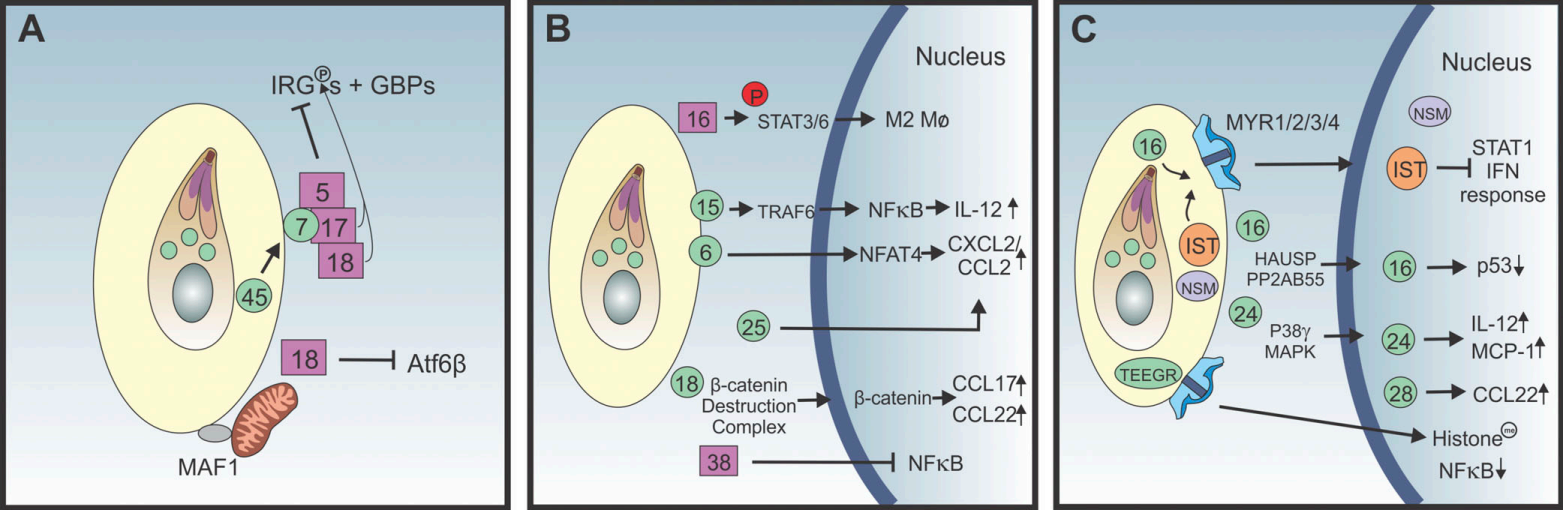
***T. gondii* effectors in immune defense. (A)** *T. gondii* effector proteins (ROP, purple squares; GRA, circles) act directly at or in the PVM to repel host Irgs, deactivate the transcription factor ATG6β, or attract mitochondria. MYR effector proteins provide a translocon for selected effector proteins, and GRA45 is a chaperone for some effectors. **(B)** *T. gondii* effector proteins act in the cytoplasm to indirectly effect transcriptional responses partly driven by NF-κB. **(C)** GRA-derived *T. gondii* nucleomodulins translocate to the nucleus and directly regulate transcriptional responses to infection. Mø, macrophage; TEEGR, *Toxoplasma* E2F4-associated EZH2-inducing gene regulator; P, phosphorylation.

The ability of *T. gondii* to create a unique nonfusogenic PV sequesters this organism from host cytosolic sensors and restricts the ability of host MHC class I to present parasite-derived peptides (Lopez et al., 2015). In addition, *T. gondii* can deploy cassettes of related effectors that target relevant host cell pathways and mediate immune evasion. These proteins, largely derived from the parasite rhoptries and dense granules, can be broadly divided into the following categories: (a) those that are present on the host cytosolic face of the PVM, where they neutralize host antimicrobial effectors (Fig. 3 A); (b) those that interface with host cell signaling pathways and modulate host cell state and migratory behavior (Fig. 3 B); and (c) those that are transported across the PVM and translocate to the host cell nucleus to modulate gene accessibility and transcriptional responses (Fig. 3 C).

### The PV interface

At the start of the invasion process, the contents of the ROP organelles, which include >40 kinases and pseudo-kinases (Peixoto et al., 2010), are secreted into the host cytosol and have a major role in protecting the surface of the PV from host effectors. Mechanistically, the ROP5 pseudo-kinases (there can be as many as 10 encoded in a given genome) act as a central scaffold to ROP17 and ROP18, which phosphorylate several IRGs and antagonize IRG recruitment to the PVM (Behnke et al., 2012; Etheridge et al., 2014; Fentress et al., 2010; Niedelman et al., 2012; Reese and Boothroyd, 2011). Interestingly, the ROP5 proteins are highly polymorphic and may be optimized for binding of a different IRG or sets of polymorphic IRGs, possibly depending on host type (Lilue et al., 2013). Indeed, each parasite strain carries its own number and type of ROP5 genes, diversifying the ROP5 repertoire as a whole. Likewise, ROP18 varies between isolates, both in sequence and in expression levels, with some strains not expressing the protein at all. The heterogeneity between parasite strains may reflect the evolutionary pressure of having a wide range of intermediate hosts in nature.

Once the PV is established, the dialogue with the host cell continues with the export of GRA proteins. Some remain within the PV, some integrate into the PVM, and some translocate across the PVM into the host cytosol, eventually reaching the host nucleus. Efficient translocation of GRA effectors across the PVM is dependent on the aspartyl protease, ASP5; ROP17; and to a lesser extent, the protein phosphatase PPM3C (Coffey et al., 2015; Hammoudi et al., 2015; Mayoral et al., 2020b; Panas et al., 2019a). The recently identified GRA45 acts as a chaperone of several parasite effectors (HRA23, MAFI, GRA5, and GRA7) on the PVM required for resistance to IRG and GBP effectors (Wang et al., 2020). GRA7 forms a complex with ROP17 and ROP18 to block IRG and GBP loading onto the PV (Alaganan et al., 2014; Hermanns et al., 2016). While the majority of ROPs and GRAs that antagonize IRG recruitment also impact GBP recruitment (Haldar et al., 2015; Selleck et al., 2013; Virreira Winter et al., 2011), others appear more specialized. For example, GRA60 dissociates IRGs from the PV but has no effect on GBPs (Nyonda et al., 2021). It is likely that export mechanisms for *T. gondii* effectors across the PVM are shared by parasite strains, while the exported proteins are specialized to interfere with divergent host resistance mechanisms. This transport mechanism may provide a viable target for drug design that would render the tachyzoite stage more susceptible to host cell antimicrobial activities.

### Targeting host cell signaling

Another strategy used by intracellular pathogens is to hijack host signaling to promote a cell state suitable for parasite growth. For *T. gondii*, this starts with the initial surface interaction with the host cell surface, which triggers epidermal growth factor receptor signaling and the FAK-Src-STAT3 pathway, which antagonize autophagic processes and preserve the nonfusogenic PV (Lopez Corcino et al., 2019; Muniz-Feliciano et al., 2013; Portillo et al., 2017). Next, the introduction of ROP effectors includes ROP18, whose N-terminus has been linked to destabilization of the ER-bound transcription factor ATF6β (Yamamoto et al., 2011) and degradation of NF-κB, which reduces the ability to produce IL-6, IL-12, and TNF (Du et al., 2014). With the establishment of the PV, the ability to export GRA proteinss provides another opportunity to interfere with signaling, and GRA18 in the host cell cytoplasm complexes with regulatory elements of the β-catenin destruction complex, which leads to an anti-inflammatory response (He et al., 2018).

Perhaps one of the best-studied parasite effectors that alter host signaling is ROP16, a polymorphic tyrosine kinase that phosphorylates and activates the host transcription factors STAT3 and STAT6 (Saeij et al., 2007). In macrophages, this promotes an M2 state characterized by an inability to make nitric oxide (NO) and low levels of GBPs and IRGs (Saha et al., 2017). Parasites that are deleted of ROP16 do not activate STAT3, resulting in increased macrophage production of IL-12, TNF, and NO (Butcher et al., 2011; Jensen et al., 2011). Interestingly, *T. gondii* can inject ROP proteins into host cells without a productive infection (Koshy et al., 2012), and the injection of ROP16 alone is sufficient to induce an M2 phenotype (Chen et al., 2020). It is notable that the loss of ROP16 results in reduced parasite growth but increased parasite-specific T cell responses, which may be explained by the ability of M2 macrophages to suppress T cell activities (Chen et al., 2020). Thus, ROP16 is a virulence factor that promotes an M2 program that contributes to a suppressive environment and limits the magnitude of parasite-specific T cell responses. This impact on cell-extrinsic activation is also apparent in an in vivo screen in pools of parasite mutants of known effector proteins that could be separated into two groups: genes required for in vivo growth within a population of mutants (such as *ROP18*, *GRA17*, and *GRA25*) and those required for growth in vivo when infecting as a single mutant, but not when part of a mutant pool (such as MYR1, MYR3, ROP17, and GRA16; Young et al., 2019). In other words, similarly to some bacterial screens, certain mutants are complemented by the presence of wild-type neighbors, whereas other mutants are intrinsically deficient and cannot be rescued by wild-type relatives. In another example, the expression of noncoding RNA miR-146a is induced by *T. gondii* and can spread to uninfected cells via exosomes (Cannella et al., 2014). Together, these observations indicate that while *T. gondii* can directly modify its host cell, it can also target those in the neighborhood that are not infected to promote an immune environment that ensures parasite survival.

Following initial encounter with a suitable host, *T. gondii* will spread from the sites of invasion to tissues such as the central nervous system (CNS), where they readily form tissue cysts, a stage critical for parasite transmission. Dissemination has been linked to the hypermotility of infected cells (Bhandage et al., 2020; Ólafsson et al., 2020), and infected monocytes display increased tethering, rolling, and adherence to vascular endothelium associated with reduced formation of integrin clusters (Harker et al., 2013). In infected cells, reduced β1 integrin activity and suppressed focal adhesion kinase phosphorylation decrease adhesion and increase migration (Cook et al., 2018). Other studies have provided evidence that the infection-induced unfolded protein response also contributes to increased migration and parasite spread (Augusto et al., 2020). These observations have now been complemented by the identification of parasite effectors that promote migration and trafficking. Thus, ROP17 increases motility and dissemination of infected monocytes, possibly through its role in the translocation of GRA effectors (Drewry et al., 2019). Similarly, the GRA protein TgWIP (*T. gondii* WAVE complex interacting protein) regulates host actin dynamics and promotes increased motility and transmigration (Sangaré et al., 2019). However, dissemination of infected cells is distinct from the ability of parasites to cross endothelial barriers to tissues such as the CNS (Courret et al., 2006; Drewry et al., 2019; Konradt et al., 2016), and in vivo screens are needed to identify parasite factors that aid this process.

### Nuclear targeting: Journey to the center of the cell

One conserved pathogen strategy to modulate host responses, first described for bacteria, is the ability to translocate effectors (nucleomodulins) to the host cell nucleus to target transcriptionally mediated events (Bierne and Pourpre, 2020). The observation that *T. gondii* interferes with chromatin remodeling events required for accessibility and activation at the TNF promoter (Leng et al., 2009) presaged our current understanding of the ability of *T. gondii* nucleomodulins (ROP16, ROP47, *Toxoplasma* E2F4-associated EZH2-inducing gene regulator, *T. gondii* inhibitor of STAT1 transcriptional activity [TgIST], *T. gondii* NCoR/SMRT modulator [TgNSM], GRA16, GRA24, GRA28, and PP2C-hn) to modify host cell functions. These proteins originate from the dense granules, and their export into the host cell is dependent on the formation of the multiprotein MYR channel within the PVM (Marino et al., 2018; Naor et al., 2018; Panas et al., 2019a). One effector, GRA16, promotes expression of genes involved in metabolism, cell cycle progression, and the p53 tumor suppressor pathway (Bougdour et al., 2013). In contrast, *Toxoplasma* E2F4-associated EZH2-inducing gene regulator (Braun et al., 2019), also known as inducer of host cyclin E (Panas et al., 2019b), translocates to the nucleus, where it induces production of EZH2, a histone-lysine *N*-methyltransferase. This enzyme participates in histone methylation that results in a nonpermissive chromatin structure and transcriptional repression for a subset of NF-κB–regulated genes such as IL-6 and IL-8 (Braun et al., 2019).

Because IFN-γ–induced STAT1 is important in control of *T. gondii*, it is counterintuitive that infection of cells alone is sufficient to induce STAT1 phosphorylation, nuclear translocation, and association with host DNA (Rosowski et al., 2014). How *T. gondii* activates STAT1 is unclear but is dependent on the ability to secrete TgIST. This GRA protein traffics to the host cell nucleus, where it recruits an Mi-2 nucleosome remodeling and deacetylase complex that act as a transcriptional repressor at sites of STAT1 binding (Gay et al., 2016; Olias et al., 2016). TgIST also binds to STAT1/STAT2 heterodimer, suppresses the type I IFN pathway, and can block IDO induction (Bando et al., 2018; Matta et al., 2019). These events provide a mechanism to actively repress STAT1-mediated transcriptional events involved in parasite control. TgNSM is another nucleomodulin that acts in concert with TgIST to block IFN-γ–mediated necroptosis (a programmed form of inflammatory cell death; Rosenberg and Sibley, 2021). It has also been proposed that the ability of *T. gondii* to block host cell cycle progression would result in reduced chromatin accessibility that would limit the host transcriptional response (Panas and Boothroyd, 2021). The ability of *T. gondii* to simultaneously repress STAT1 and NF-κB activities while allowing sustained STAT3/6 and p53 transcriptional effects and modifying chromatin accessibility identifies the host cell nuclear landscape as a key battleground for parasite survival.

## Latency and subversion

A key feature of the lifecycle of *T. gondii* is its ability to respond to cellular stress, transform to the slow replicating bradyzoite, and form tissue cysts. This developmental stage is critical for parasite persistence and oral transmission, and it is thought that low levels of reactivation of the latent form contribute to sustained immune activation. Cyst formation can occur in many cell types in vitro*,* but in the CNS, cysts are almost exclusively found in neurons (Cabral et al., 2016), which may contribute to immune evasion. The ability of cysts to persist in neurons may reflect the long-lived nature of these cells and their expression of low levels of MHC class I and reduced IFN-γ signaling (Klein and Hunter, 2017). However, the loss of MHC class I on neurons results in reduced ability to control tachyzoites (Salvioni et al., 2019), and in human neurons, an IDO1-dependent activity limits growth of *T. gondii* (Bando et al., 2019). In addition, the use of *T. gondii* that secrete Cre into host cells identified the presence of uninfected neurons in vivo that had interacted with *T. gondii* (Cabral et al., 2020; Cabral et al., 2016; Mendez et al., 2018). These results indicate that in vivo neurons can clear *T. gondii,* but whether this is due to the ability to target tachyzoite or bradyzoite stages is uncertain. The finding that the loss of STAT1 in astrocytes in vivo results in cyst formation in these cells (Hidano et al., 2016) suggests that IFN-γ can promote bradyzoite control in nonneuronal cell types. Furthermore, the ability of bradyzoites to use TgMSN and TgIST to block IFN-γ–mediated necroptosis (Rosenberg and Sibley, 2021) implies the need for the cyst stage to evade IFN-γ–mediated activity.

While long considered to be inert, it is now recognized that bradyzoites undergo episodic bursts of proliferation, internalize host-derived macromolecules, and export parasite effectors (Kannan et al., 2021; Watts et al., 2015). The cyst wall is an important interface between host and parasite that sits just below the PVM (Guevara et al., 2020; Tu et al., 2020; Young et al., 2020), and not every protein that can escape from the PVM in tachyzoites can cross the cyst wall (Krishnamurthy and Saeij, 2018). Nevertheless, the ability of some effectors to cross the cyst wall indicates the presence of a transport mechanism (Mayoral et al., 2020a; Paredes-Santos et al., 2019; Seizova et al., 2019 *Preprint*; Tomita et al., 2021). For example, the GRA proteins TgIST and TgMSN are secreted from cysts and block STAT1-mediated activities (Mayoral et al., 2020a; Rosenberg and Sibley, 2021). There are also other GRA and ROP proteins (GRA2, 3, 7, 8, 9, 12, or 14; MAG1; and ROP21/27) that are not required for parasite growth or ability to differentiate into cysts in vitro, but their individual deletion results in reduced cyst formation in vivo (Fox et al., 2019; Fox et al., 2016; Jones et al., 2017; Tomita et al., 2021). There are multiple possible interpretations of these observations, and this parasite interface with neurons is not well understood, in large part because of a paucity of molecular tools to study cyst biology. The identification of BFD1, a Myb-like transcription factor, as a lineage-defining regulator of the bradyzoite transcriptional program (Waldman et al., 2020) provides an opportunity to identify parasite effectors that may be uniquely relevant to cyst immune evasion and to start testing the importance of parasite latency on infection outcome.

## Future directions

The research community that studies *T. gondii* has applied the full range of genetic and biochemical approaches to understand how host cells detect and limit parasite replication and how *T. gondii* counters these processes. These studies have helped to identify evolutionarily conserved strategies (e.g., targeting the STAT proteins) relevant to other intracellular pathogens and have provided insight into core cellular processes required to limit growth of intracellular pathogens. The continued development of novel tools to manipulate *T. gondii* (Sangaré et al., 2019; Wang et al., 2020) and the application of artificial intelligence and high-resolution imaging in vitro (Fisch et al., 2019b) and in vivo (Coombes and Robey, 2010) have already provided opportunities to better understand this host–pathogen interaction. With advances in understanding the molecular basis for different forms of cell death, perhaps *T. gondii* provides a model to explore the general topic of how host cell death influences innate and adaptive responses to pathogens. There are already indications that this cellular machinery intersects with sensing of *T. gondii*, but whether this is important for the processing and presentation of parasite antigens is an open question (Lee et al., 2015).

The identification of host mechanisms that limit replication of *T. gondii* is balanced by the discovery of *T. gondii* effectors that modulate host biology. Many of these advances have relied on a relatively limited number of host (mouse and human) models, while differences in parasite strains led to the identification of GRA15, ROP16, and ROP18 as key effectors that affect virulence. Nevertheless, strain-specific differences that are not easily explained may provide new insights into the host pathways that target *T. gondii*. For example, some parasite strains appear to engage endosomal TLR or the cytoplasmic receptor retinoic acid-inducible gene 1 to induce type I IFNs, but the basis for this differential activity is unclear (Melo et al., 2013). Likewise, the polymorphisms associated with the ROP and GRA proteins and their links with virulence (e.g., the ROP5 isoforms associated with hypervirulence of the South American strain; Lilue et al., 2013) suggest they are under immune pressure. This ongoing host–pathogen dialogue also promotes host adaptation, and important IRG polymorphisms in wild-derived Eurasian mice determine the ability to control *T. gondii* (Lilue et al., 2013; Murillo-León et al., 2019). Thus, an emphasis on additional strain and host combinations (e.g., the zebrafish; Yoshida et al., 2020) has the potential to identify unique evasion strategies and provide additional insights into the processes that lead to the control of vacuolar pathogens.
